# Racial inequity and other social disparities in the diagnosis and management of bladder cancer

**DOI:** 10.1002/cam4.4917

**Published:** 2022-06-08

**Authors:** Shaakir Hasan, Stanislav Lazarev, Madhur Garg, Keyur Mehta, Robert H. Press, Arpit Chhabra, J. Isabelle Choi, Charles B. Simone, Daniel Gorovets

**Affiliations:** ^1^ The New York Proton Center New York New York USA; ^2^ Montefiore Medical Center, Department of Radiation Oncology Bronx New York USA; ^3^ Mount Sinai Medical Center, Department of Radiation Oncology New York New York USA; ^4^ Memorial Sloan Kettering Cancer Center, Department of Radiation Oncology New York New York USA

**Keywords:** black, bladder cancer, disparities, inequities, race, social disparities

## Abstract

**Background:**

We investigate the impact of gender, race, and socioeconomic status on the diagnosis and management of bladder cancer in the United States.

**Methods:**

We utilized the National Cancer Database to stratify cases of urothelial cell carcinoma of the bladder as early (Tis, Ta, T1), muscle invasive (T2–T3, N0), locally advanced (T4, N1–3), and metastatic. Multivariate binomial and multinomial logistic regression analyses identified demographic characteristics associated with stage at diagnosis and receipt of cancer‐directed therapies. Odds ratios (OR) are reported with 95% confidence intervals.

**Results:**

After exclusions, we identified 331,714 early, 72,154 muscle invasive, 15,579 locally advanced, and 15,161 metastatic cases from 2004–2016. Relative to diagnosis at early stage, the strongest independent predictors of diagnosis at muscle invasive, locally advanced, and metastatic disease included Black race (OR = 1.19 [1.15–1.23], OR = 1.49 [1.40–1.59], OR = 1.66 [1.56–1.76], respectively), female gender (OR = 1.21 [1.18–1.21], OR = 1.16 [1.12–1.20], and OR = 1.34 [1.29–1.38], respectively), and uninsured status (OR = 1.22 [1.15–1.29], OR = 2.09 [1.94–2.25], OR = 2.57 [2.39–2.75], respectively). Additional demographic factors associated with delayed diagnosis included older age, treatment at an academic center, Medicaid insurance and patients from lower income/less educated/more rural areas (all *p* < 0.01).

Treatment at a non‐academic center, older age, women, Hispanic and Black patients, lower income and rural areas were all less likely to receive cancer‐directed therapies in early stage disease (all *p* < 0.01). Women, older patients, and Black patients remained less likely to receive treatment in muscle invasive, locally advanced, and metastatic disease (all *p* < 0.01).

**Conclusion:**

Black race was the strongest independent predictor of delayed diagnosis and substandard treatment of bladder cancer.

## Graphical abstract

In a robust multivariable analysis of the national cancer database, among demographic characteristics we found that black patients were most significantly, consistently, and independently associated with both late diagnosis and use of non‐standard therapies.

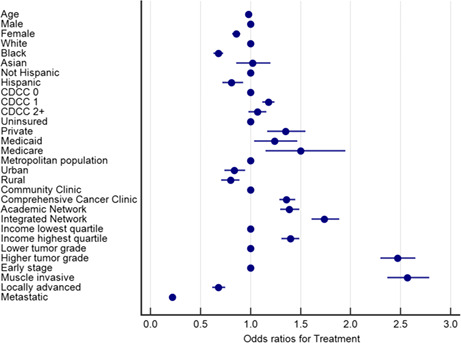

## 1 INTRODUCTION

Affecting over 80,000 Americans per year and causing 20,000 annual deaths, bladder cancer is the sixth most common malignancy in America.^1^, ^2^ Fortunately, it can present early and is often curable. Not all demographic groups, however, have the same rate of early detection. Data from the latter decades of the 20th century indicate that both women and Black patients (independent of gender) are typically diagnosed in later stages.^3^ Consequently, both Black and female patients have worse long‐term survival compared to their white, male counterparts.^4^ This remains the case even though women were shown to be no less likely to receive the optimal treatment in muscle‐invasive bladder cancer.^5^ While not the main endpoint, the same study suggested that such equitable treatment between genders may not extend to racial disparities, although this is currently not well described.

Over the past several years, organizations like the American Society of Clinical Oncology have attempted to raise awareness regarding health disparities in cancer care.^6^, ^7^ Perhaps because of this emphasis, a plethora of recent publications have emerged, highlighting various demographic inequities in the most common cancers.^8^, ^9^, ^10^, ^11^, ^12^, ^13^, ^14^ Studies have shown that Black patients are often diagnosed later, are less likely to receive optimal treatment, and wait longer to be treated for a multitude of conditions.^15^, ^16^, ^17^ Disparities in bladder cancer, particularly with respect to race, remains relatively absent in the literature. Therefore, in this study we analyzed the largest contemporary American cancer database to investigate how racial, gender, and socioeconomic disparities relate to the diagnosis and management of urothelial carcinoma of the bladder. Our hypothesis is that racial disparities are the strongest predictors of inequitable bladder cancer detection, tumor‐directed therapies, and treatment delays.

## 2 METHODS

The National Comprehensive Cancer Network (NCCN) guidelines were used to risk‐stratify bladder cancer patients based on management options: non‐muscle invasive disease (early stage), muscle invasive disease, locally advanced disease, and metastatic disease.^18^ We then evaluated the association of race, among other covariate demographic characteristics, with three specific endpoints: stage at diagnosis, type of treatment delivered, and time interval from diagnosis to treatment. The National Cancer Database (NCDB) is overseen by the American College of Surgeons and the Commission on Cancer and encompasses an estimated 70% of annual newly diagnosed cancer cases in the United States. Given its retrospective nature and de‐identified dataset, this study was exempt from institutional review board approval. The American College of Surgeons and the Commission on Cancer have not verified and are not responsible for the analytic or statistical methodology employed, or the conclusions drawn from these data.

We queried the NCDB from 2004–2016 for all patients with a diagnosis of urothelial carcinoma of the bladder. From an initial file of 577,675 patients, cases were excluded if any of the following variables from each patient were unknown: race (*n* = 75,455), insurance (*n* = 7396), income and education (*n* = 2292), population density (*n* = 12,239), non‐urothelial cell histology (*n* = 10,409), and stage (*n* = 35,276). Ultimately, 434,608 cases remained for this analysis and were further stratified into early stage (*n* = 331,714), muscle invasive (*n* = 72,154), locally advanced (*n* = 15,579), and metastatic (*n* = 15,161) cohorts. Early stage included Tis/Ta/T1 N0 M0, muscle invasive included T2–T3 N0 M0, locally advanced included T4 or N1–3 M0, and metastatic included M1 disease.

These four groups formed the basis of our multinomial multivariate logistic regression analysis, in which we calculated the conditional probability of being diagnosed in a later stage (muscle invasive, locally advanced, or metastatic) relative to early stage for various demographic characteristics. For instance, with racial disparities “early stage” and “Asian” were used as the reference values, and we then reported the probability of being diagnosed with muscle invasive disease as white patients relative to an Asian patients, locally advanced disease relative to an Asian patient, and metastatic disease relative to an Asian patient. The same probabilities were then tabulated for black patients relative to Asian patients, and this process was repeated for the following demographic characteristics: age (as a continuous variable), gender, Hispanic ethnicity, Charlson Deyo Comorbidity (CDCC) score, insurance, education, population density, type of treatment facility, education level, income, and tumor grade. The multinomial multivariable logistic regression model allowed for the control of covariability of all the aforementioned factors while still investigating a non‐binomial endpoint such as stage at diagnosis. Because of the large nature of the data set, factors significant on univariable analysis were entered using a stepwise backward elimination process. Adjusted odds ratios (OR) and 95% confidence intervals (CI) are reported, using an α level of 0.05 to indicate statistical significance.

The second part of our analysis explored the association between the aforementioned demographic characteristics and treatment received using a binomial multivariable regression analysis. Since the acceptable standard treatment varies considerably by risk group, this analysis was performed within each group. “Acceptable treatment” was defined as either a transurethral resection of bladder tumor (TURBT) (with or without adjuvant therapy) or cystectomy in the early stage group, definitive chemoradiation or cystectomy (with or without systemic therapy) in the muscle invasive group, cystectomy and systemic therapy or radiotherapy and systemic therapy in the locally advanced group, and systemic therapy in the metastatic group. As the focus of this study is race, we then performed the same binomial multivariable regression analysis for each group, but propensity‐matched Black and non‐Black patients to account for indication bias. Multivariable logistic regression was used to calculate a propensity score indicative of conditional probability of being a Black patient (or not) within the data set. The propensity model included observable variables associated with either race on multivariable logistic regression. A Cox proportional hazards model was then constructed incorporating the propensity score but also excluding factors included in the propensity score calculation to avoid overcorrection. The assumption of balance was additionally validated by stratifying the data into propensity score–based quintiles and confirming that the difference in propensity score mean per quintile was <0.10. The final analysis was to perform a similar calculation but using “time from diagnosis to treatment” (greater or less than the median value for each risk group) as the statistical endpoint. Statistics were performed via MedCalc version 19.1 and SPSS version 24.

The NCDB contains various patient‐related and socioeconomic factors ranging from race, median household income, type of insurance, gender, age, education level, distance from treatment facility, and comorbidity score. Race was simplified into three categories: Caucasian, African American, or Asian. For binomial regression analysis it was further coded into “Black” or “non‐Black.” The Charlson/Deyo comorbidity index was recorded and quantified the degree of comorbidities in the patient population. Socioeconomic data in the patients' residence census tract were divided into quartiles based upon the percentage of persons with less than a high school education and median household income based on zip code of residence. Facility type was grouped according to the Commission on Cancer accreditation category (community cancer center, comprehensive community cancer center, and academic/research program). Locations were described based on data provided by the United States Department of Agriculture Economic Research Service. Insurance status is documented in the NCDB as it appears on the admission page.

## 3 RESULTS

### 3.1 Patient characteristics

A clinical and demographic breakdown of patients diagnosed from 2004–2016 included in the early stage (*n* = 331,714), muscle invasive (*n* = 72,154), locally advanced (*n* = 15,579), and metastatic groups (*n* = 15,161) are detailed in Table 1. Among all patients, 5.2%, 47.2%, 23.9%, 13.9%, 2.7%, and 2.0% presented with Tis, Ta, T1, T2, T3, and T4 disease, respectively. Positive nodes were detected in 2.8% of cases and 3.5% presented with metastasis. All patients had transitional cell histology and 36.2% of cases were either well or moderately differentiated, whereas 46.6% were poorly or undifferentiated (remainder were unknown). Median age across the four cohorts was 72 years with an interquartile range of 64–80. The Northeast region contributed 24.7% of cases, 20.7% came from the Southeast, 32.5% from the Midwest, 10.2% from the Southwest, and 11.0% from the West coast.

**TABLE 1:** Disparities in bladder cancer diagnosis

Demographic parameter	Early stage	Muscle invasive	Locally advanced	Metastatic
Gender				
Male	252,434 (77.2%)	52,309 (16.0%)	11,395 (3.5%)	10,640 (3.3%)
Female	79,280 (73.5%)	19,845 (18.4%)	4184 (3.9%)	4521 (4.2%)
Race				
White	310,390 (76.8%)	66,302 (16.4%)	13,936 (3.4%)	13,420 (3.3%)
Black	15,763 (67.8%)	4673 (20.1%)	1365 (5.9%)	1458 (6.3%)
Asian	5561 (76.2%)	1179 (16.1%)	278 (3.8%)	283 (3.9%)
Hispanic ethnicity				
Yes	8872 (74.7%)	1959 (16.5%)	524 (4.4%)	523 (4.4%)
No	322, 842 (76.4%)	70,195 (16.6%)	15,055 (3.6%)	14,638 (3.3%)
CDCC Score				
0	235,792 (77.3%)	48,091 (15.8%)	10,695 (3.5%)	10,261 (3.4%)
1	67,783 (75.0%)	16,201 (17.9%)	3280 (3.6%)	3131 (3.5%)
2	20,046 (72.0%)	5478 (19.7%)	1140 (4.1%)	1183 (4.2%)
3	8093 (70.2%)	2384 (20.7%)	464 (4.0%)	586 (5.1%)
Population setting				
Rural	12,508 (71.9%)	3465 (19.9%)	750 (4.3%)	677 (3.9%)
Urban	35,886 (73.4%)	9174 (18.8%)	2043 (4.2%)	1783 (3.6%)
Metropolitan	283,320 (76.9%)	59,515 (16.2%)	12,786 (3.5%)	12,701 (3.4%)
Insurance				
Uninsured	5595 (67.6%)	1547 (18.7%)	544 (6.6%)	586 (7.2%)
Private	97,829 (79.5%)	17,879 (14.5%)	3872 (3.1%)	3509 (2.9%)
Medicaid	8404 (64.5%)	2621 (20.1%)	935 (7.2%)	1061 (8.1%)
Medicare	217,024 (75.8%)	49,327 (17.2%)	10,036 (3.5%)	9793 (3.4%)
Income				
Lowest Quartile	44,330 (71.9%)	11,800 (19.1%)	2789 (4.5%)	2722 (4.4%)
Second Quartile	73,007 (74.2%)	17,654 (17.9%)	3942 (4.0%)	3755 (3.8%)
Third Quartile	91,664 (76.5%)	19,945 (16.6%)	4223 (3.5%)	4022 (3.4%)
Highest Quartile	122,713 (79.3%)	22,755 (14.7%)	4625 (3.0%)	4662 (3.0%)
Treatment facility				
Community Cancer	40,700 (80.1%)	6964 (13.7%)	1394 (2.7%)	1728 (3.4%)
Comprehensive Community	154,087 (79.0%)	28,977 (14.9%)	5753 (2.9%)	6271 (3.2%)
Academic	87,240 (69.3%)	27,019 (21.5%)	6560 (5.2%)	5011 (4.0%)
Integrated Network	46,526 (76.2%)	8903 (16.7%)	1757 (3.6%)	2059 (3.5%)

Abbreviation: CDCC, Charlson Deyo Comorbidity score.

### 3.2 Stage at diagnosis

A majority of cases were diagnosed at an early stage, regardless of demographic factor. The proportion of white patients diagnosed with early, muscle invasive, locally advanced, and metastatic disease were 76.8%, 16.4%, 3.4%, and 3.3%, respectively, compared to 67.8%, 20.1%, 5.9%, and 6.3% for Black patients (*p* < 0.001). There were several demographic characteristics independently associated with diagnosis at a more advanced stage per multivariable multinomial regression analysis, including female gender, Black race, a higher CDCC comorbidity score, lack of insurance, lesser populated counties, regions with lower median income and lower education, and treatment at an academic center. The corresponding odds ratios with 95% CIs are detailed completely in Table 2.

**TABLE 2:** Multivariable multinomial regression analysis for stage at diagnosis relative to early stage expressed as odds ratios (95% confidence interval in parentheses)

	Early Stage (reference)	Muscle Invasive	Locally Advanced	Metastatic
Age (continuous)	1	**1.011(1.010–1.012)**	**0.994(0.992–0.995)**	0.998(0.997–1.00)
Gender				
Female	1	—	—	—
Male	—	**0.829 (0.81–0.84)**	**0.862 (0.83–0.90)**	**0.747 (0.72–0.77)**
Race				
Asian	1	—	—	—
White	—	0.959(0.90–1.02)	0.986(0.87–1.12)	1.057(0.94–1.20)
Black	—	**1.191(1.15–1.23)**	**1.492(1.40–1.59)**	**1.66(1.56–1.76)**
Hispanic				
Yes	1	—	—	—
No	—	1.055(1.0–1.11)	0.931(0.85–1.02)	**0.905(0.82–0.99)**
CDCC Score				
0	1	—	—	—
1	—	**1.163(1.14–1.19)**	**1.402(1.33–1.48)**	**1.057(1.01–1.10)**
≥2	—	**1.323(1.21–1.37)**	**1.458(1.25–1.58)**	**1.348(1.27–1.44)**
Insurance				
Medicare	1	—	—	—
Medicaid	—	**1.294(1.23–1.36)**	**1.969(1.79–2.16)**	**2.285(2.09–2.50)**
Private	—	**0.823(081–0.84)**	**0.857(0.83–0.89)**	**0.812(0.78–0.85)**
Uninsured	—	**1.221(1.15–1.29)**	**2.093(1.94–2.25)**	**2.566(2.39–2.75)**
County (by population)				
Metropolitan (≥20 k)	1	—	—	—
Urban (2.5 k–20 k)	—	**1.173(1.14–1.20)**	**1.198(1.14–1.26)**	1.039(0.95–1.10)
Rural (<2.5 k)	—	**1.227(1.18–1.28)**	**1.219(1.13–1.32)**	**1.103(1.02–1.20)**
Median income				
Highest Quartile	1	—	—	—
Third Quartile	—	**1.15(1.12–1.17)**	**1.15(1.10–1.21)**	1.05(1.0–1.10)
Second Quartile	—	**1.23(1.20–1.26)**	**1.27(1.20–1.34)**	**1.15(1.09–1.21)**
Lowest Quartile		**1.26(1.22–1.30)**	**1.26(1.18–1.35)**	**1.16(1.08–1.24)**
Facility				
Integrated Network	1	—	—	—
Community Center	—	**0.827(0.80–0.86)**	**0.828(0.77–0.89)**	**0.913(0.86–0.98)**
Comprehensive Community	—	**0.955(0.93–0.98)**	**0.969(0.92–1.02)**	**0.922(0.88–0.97)**
Academic	—	**1.619(1.58–1.66)**	**1.912(1.81–2.02)**	**1.236(1.17–1.30)**
Tumor Grade				
Undifferentiated	1	—	—	—
Poorly differentiated	—	**1.10(1.08–1.12)**	**1.14(1.10–1.18)**	**1.14(1.10–1.19)**
Moderately differentiated	—	**0.087(0.08–0.09)**	**0.104(0.09–0.11)**	**0.08(0.07–0.09)**
Well differentiated	—	**0.031(0.03–0.033)**	**0.042(0.04–0.05)**	**0.04(0.03–0.043)**

*Note*: Bold indicates statistical significance.

Abbreviation: CDCC, Charlson Deyo Comorbidity score.

Older patients (with age as a continuous variable) were more likely to be diagnosed with muscle invasive disease relative to early stage (OR = 1.011, 95% CI 1.010–1.012), but less likely to be diagnosed with locally advanced or metastatic disease (OR = 0.994, 95% CI 0.992–0.995). There was also a clear separation with tumor grade and stage at diagnosis, as well/moderately differentiated tumors were between 9.6 and 32.3 times more likely to be diagnosed at an early stage relative to poorly or undifferentiated tumors (*p* < 0.001). Among the demographic correlates of diagnosis at a later stage, only three demonstrated a progressively stronger correlation as disease advanced: patients with Medicaid or without insurance (relative to Medicare and private insurance), and Black patients (relative to both white and Asian patients). Uninsured patients were 1.22, 2.09, and 2.57 times more likely to be diagnosed with muscle invasive, locally advanced, and metastatic disease, respectively (all *p* < 0.001), and the odds ratios for Medicaid patients were 1.29, 1.97, and 2.29, respectively (all *p* < 0.001). Black patients were more likely to be diagnosed with muscle invasive (OR = 1.19, 95% CI 1.15–1.23), locally advanced (OR = 1.49, 95% CI 1.40–1.59), or metastatic disease (OR = 1.66, 95% CI 1.56–1.76) relative to white or Asian patients (all *p* < 0.001).

### 3.3 Treatment selection

Within the early stage cohort, 54% of patients with carcinoma in situ received a TURBT without adjuvant therapy, 25.2% received a TURBT with adjuvant therapy (predominantly intravesicular BCG), and 7.3% underwent a cystectomy. Ta lesions were treated by TURBT alone, TUBRT and adjuvant therapy, and cystectomy in 72.6%, 17.9%, and 1.3% of cases, respectively. TURBT was also the most common treatment for T1 disease (87.2%), followed by cystectomy (9.2%), and nearly half of the T1 lesions were also treated with adjuvant therapy (48.3%). Muscle invasive disease was treated with TURBT alone, TURBT and chemotherapy, TURBT and radiotherapy (+/− chemotherapy), cystectomy alone, and cystectomy and chemotherapy (+/− radiotherapy) in 23.0%, 10.3%, 15.2%, 25.1%, and 26.4% of treated cases, respectively. For locally advanced disease those proportions were 16.3%, 17.0%, 15.3%, 15.2%, and 31.4%, respectively, in addition to 4.7% of patients who were treated with chemotherapy alone. Lasty, metastatic disease was treated with radiotherapy +/− chemotherapy in 21.9% of cases, chemotherapy alone in 54.2% of cases, and with palliative intent in 9.3% (remainder not reported).

**FIGURE 1:**
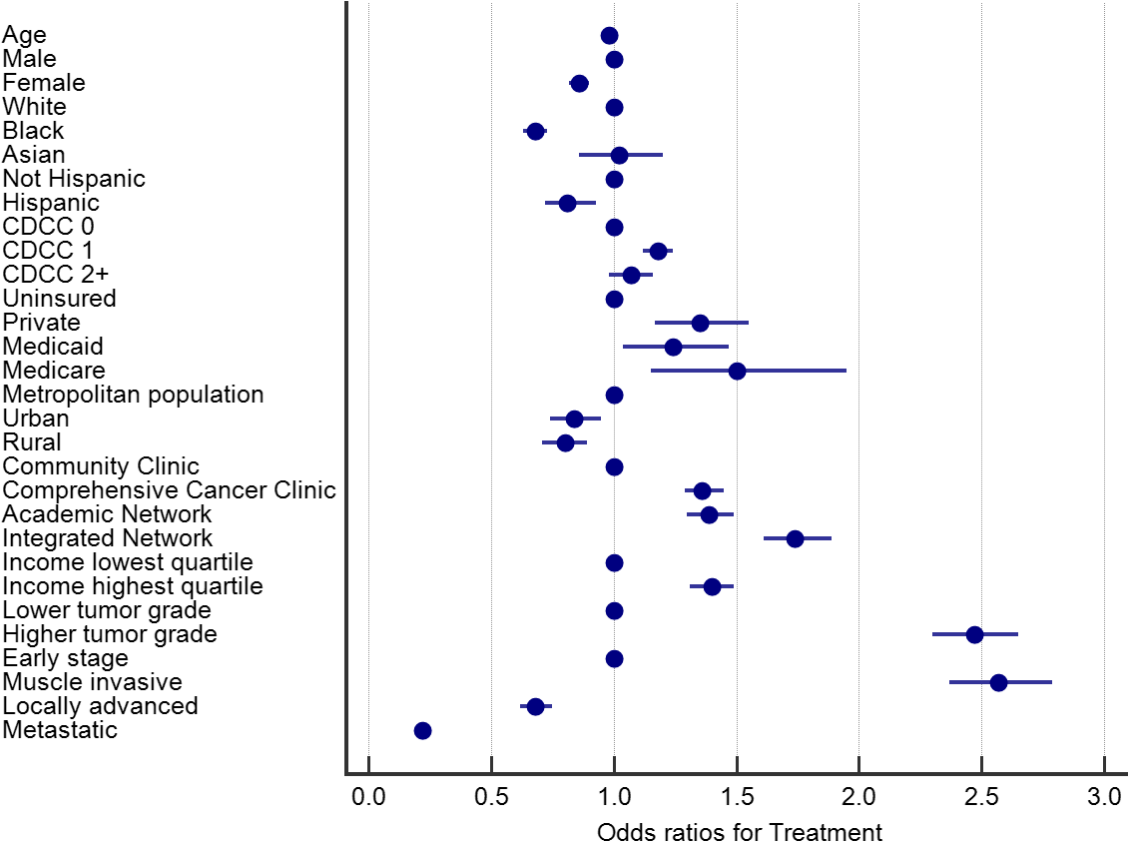
Forest plot for “acceptable” treatment received for all comers per multivariable binomial regression analysis. Age is a continuous variable. CDCC, Charlson Deyo Comorbidity score

Among all early stage cases, a slightly greater proportion of non‐Black patients received an NCCN‐listed treatment (97.8% compared to 96.6%), but the distribution of treatment modality was similar between races among those who received some type of therapy. Only 0.8% of muscle invasive disease did not receive an NCCN‐recommended treatment among non‐Black patients compared to 1.7% among Black patients. For locally advanced disease, 3.2% of non‐Black patients did not receive a recommended treatment compared to 5.0% of Black patients. In the metastatic setting, 9.0% of non‐Black patients received palliative/supportive therapy only, compared to 11.3% of Black patients (all *p* < 0.01). Table 3 depicts a complete list of odds ratios reflecting the independent likelihood of each demographic characteristic receiving tumor‐directed therapy for each prognostic group per multivariable bimomial regression analysis, and Figure 1 illustrates these associations as a forest plot for all groups combined.

**TABLE 3:** Odds ratios (95% confidence interval) for cancer‐directed therapy by stage per multivariable binomial regression analysis

	Early stage	Muscle invasive	Locally advanced	Metastatic
Age (continuous)	**0.98 (0.98–0.99)**	**0.96 (0.95–0.97)**	**0.96 (0.95–0.97)**	**0.97 (0.96–0.98)**
Gender				
Male (reference)	1	1	1	1
Female	**0.87 (0.83–0.92)**	**0.83 (0.70–0.98)**	**0.74 (0.61–0.89)**	**0.88 (0.78–0.99)**
Race				
White (reference)	1	1	1	1
Black	**0.67 (0.61–0.74)**	**0.51 (0.39–0.66)**	**0.64 (0.48–0.86)**	**0.78 (0.65–0.95)**
Asian	1.01(0.90–1.34)	0.71(0.40–1.27)	1.03(0.51–2.12)	0.87(0.58–1.31)
Black (propensity matched)	**0.68 (0.58–0.77)**	**0.53 (0.34–0.69)**	**0.65(0.48–0.88)**	**0.79(0.62–0.97)**
Hispanic				
No	1	1	1	1
Yes	**0.85 (0.74–0.97)**	0.74(0.48–1.14)	0.76(0.47–1.21)	**0.73 (0.55–0.97)**
CDCC score				
0	1	1	1	1
1	**1.23(1.16–1.31)**	1.15(0.94–1.39)	1.23(0.97–1.55)	0.89(0.78–1.03)
2	**1.13(1.02–1.26)**	1.19(0.87–1.63)	0.94(0.68–1.30)	**0.80(0.66–0.98)**
Insurance				
Uninsured	1	1	1	1
Private	**1.37(1.16–1.63)**	1.47(0.84–2.58)	**1.96(1.10–3.50)**	**1.61(1.17–2.21)**
Medicaid	**1.34(1.07–1.67)**	1.60(0.77–3.29)	0.74(0.40–1.38)	1.09(0.76–1.56)
Medicare	**1.54(1.30–1.83)**	1.75(1.00–3.06)	1.42(0.81–2.46)	**1.55(1.14–2.45)**
Population				
Metropolitan	1	1	1	1
Urban	**0.77(0.67–0.90)**	0.99(0.65–1.52)	1.12(0.67–1.88)	1.02(0.74–1.39)
Rural	**0.75 (0.65–0.86)**	0.92(0.62–1.35)	0.88(0.55–1.40)	0.99(0.75–1.31)
Facility				
Community	1	1	1	1
Comprehensive	**1.42(1.33–1.52)**	**1.27(1.01–1.60)**	1.10(0.81–1.49)	1.10(0.92–1.32)
Academic	**1.38(1.28–1.49)**	**2.14(1.65–2.78)**	1.32(0.96–1.81)	1.00(0.83–1.22)
Integrated Network	**1.88(1.70–2.06)**	**1.79(1.30–2.45)**	1.27(0.87–1.87)	1.19(0.94–1.49)
Income				
Lowest Quartile	1	1	1	1
Second Quartile	**1.22(1.13–1.32)**	1.08(0.85–1.36)	1.21(0.92–1.60)	1.00(0.84–1.19)
Third Quartile	**1.43(1.33–1.55)**	**1.43(1.11–1.82)**	1.30(0.98–1.71)	1.16(0.97–1.38)
Highest Quartile	**1.45(1.43–1.56)**	**1.37(1.08–1.75)**	1.30(0.98–1.72)	**1.26(1.05–1.51)**
T stage^a^				
Lower	1	1	1	1
Higher	**2.47(2.28–2.67)**	0.82(0.70–1.10)	**1.62(1.33–1.98)**	1.04 (0.78–1.64)
Grade^b^				
Lower	1	1	1	1
Higher	**2.58(2.26–2.94)**	**1.59(1.28–1.97)**	**3.02(2.42–3.78)**	**3.37(2.94–3.86)**

*Note*: Bold indicates statistical significance.

Abbreviation: CDCC, Charlson Deyo Comorbidity score.

^a^ Tis versus T1‐Ta for early stage, T1 versus T2–T3 for muscle invasive, T1–3 versus T4 for locally advanced and metastatic.

^b^ Lower grade is well or moderately differentiated, higher grade is poorly or undifferentiated.

Pathologic characteristics such as higher tumor grade correlated with a greater likelihood of receiving treatment overall (OR = 2.47, 95% CI 2.30–2.65), as did advanced T stage in early (OR = 2.47, 95% CI 2.28–2.67) and locally advanced disease (OR = 1.62, 95% CI 1.33–1.98). Several demographic parameters independently correlated with statistically different likelihoods for receiving standard therapies; however, only female gender and Black race were less likely to receive an NCCN‐recommended treatment for every risk group. The odds ratios for women receiving treatment (relative to men) in early stage, muscle invasive, locally advanced, and metastatic disease was 0.87 (95% CI 0.83–0.92), 0.83 (95% CI 0.70–0.98), 0.74 (0.61–0.89), and 0.88 (0.78–0.99), respectively. The odds ratios were more pronounced for Black patients (men and women), at 0.67 (95% CI 0.61–0.74) for early stage, 0.51 (95% CI 0.39–0.66) for muscle invasive, 0.64 (95% CI 0.48–0.86) for locally advanced, and 0.78 (95% CI 0.65–0.95) for metastatic disease. After propensity matching for race, the odds ratios were effectively unchanged (+/− 0.02). In addition to white race and male gender, patients were more likely to receive an NCCN‐listed therapy if they were non‐Hispanic, had private insurance or Medicare, lived in a more population‐dense region, were treated outside of a community clinic, had higher income, or had muscle invasive disease (all *p* < 0.01).

### 3.4 Time to treatment

The median intervals from diagnosis to treatment were 19 (IQR 7–35), 23 (IQR 13–43), 25 (IQR 10–47), and 15 (IQR 6–33) days for early, muscle‐invasive, locally‐advanced, and metastatic disease, respectively. In another multivariable binomial regression analysis, which demographic factors correlated with receiving treatment either before or after the median value within each risk group were investigated. Except for treatment at a community cancer clinic and in rural areas, no other demographic characteristics correlated with time from diagnosis to treatment, including race or gender. Treatment at a community center was associated with longer time to treatment for early stage (OR = 1.25, 95% CI 1.21–1.29), muscle invasive (1.28, 95% CI 1.18–1.39), and locally advanced disease (OR = 1.19, 95% CI 1.03–1.38), whereas there was no such association in metastatic disease. Similarly, treatment in rural areas associated with longer time to treatment for early stage (OR = 1.28, 95% CI 1.22–1.34) and muscle invasive disease (OR = 1.14, 95% CI 1.05–1.24), but there was no association for locally advanced or metastatic disease.

## 4 DISCUSSION

Almost 15 years ago, Lee et al. published a 30‐year SEER analysis of bladder cancer patients treated across three decades from 1973–1999 revealing that Black patients presented at a later stage than white patients.^3^ Lee's analysis reported a relatively consistent proportion of African Americans diagnosed in both the early (65%–68%) and metastatic stages (5%–8%) compared to white patients, who were more likely to be diagnosed with localized disease (78%–80%) and less likely to be diagnosed with metastatic disease (2%–3%). Despite capturing more contemporary data (2004–2016), our study mirrors the previous analysis, with white patients diagnosed in early stage and metastatic bladder cancer in 76.8% and 3.3% of cases, compared to 67.8% and 6.3% for Black patients.

With the exception of uninsured/Medicaid patients, Black race was the strongest independent predictor of diagnosis at a later stage, and this discrepancy widened as stage advanced. The mechanism behind this disparity is not well studied and likely results from a combination of biologic and social factors. For instance, the prior SEER analysis revealed that Black patients were more likely to have higher grade tumors, and that may have factored into presenting at later stages.^3^ That correlation was evident in our analysis as well; however, unlike the SEER study, we accounted for grade in our multivariable logistic regression model and the racial disparity remained. As such, it is likely that social factors also contribute to this disparity. Distrust of the medical community by some Black patients, a reduced effort by physicians to work up Black patients, and an overall lack of access to healthcare among Black patients have been reported in other cancers, and those factors may also contribute to the disproportionately advanced stage at diagnosis among Black patients in this current analysis.^19^, ^20^, ^21^

In addition to race, access to healthcare was the only other demographic factor independently associated with diagnosis at a later stage. More specifically, patients without insurance were over 3 times more likely to be diagnosed with metastatic disease compared to those with private insurance, which was the single strongest predictor of late stage diagnosis of all demographic factors assessed. This is not surprising, as uninsured patients often present in later stages of disease regardless of the ailment^22^, ^23^, ^24^; however, these findings highlight the intersectionality of healthcare access/coverage with racial disparities and suggest the latter may not be mitigated without addressing the former, as argued by Buchmueller and colleagues in their analysis of the Affordable Care Act and its impact on the racial disparity gap in healthcare.^25^

Other correlates of diagnosis at a later stage include women, higher grade (the strongest non‐demographic predictor), higher comorbidity score, and treatment in rural areas. The later presentation and worse prognosis of bladder cancer in women is well established in the literature and may be the result of mistaking the early signs and symptoms of bladder cancer, such as hematuria, for other more common ailments such as postmenopausal bleeding and urinary tract infections.^5^, ^26^ Unexpectedly, neither income nor level of education proved to be predictors of diagnosis at a particular stage, when all other factors were controlled. It may also be surprising to learn that treatment at an academic institution, compared to a community cancer clinic, was more likely to result in diagnosis at an advanced stage. This may be due to referral patterns, as patients presenting with signs and symptoms of advanced disease were probably more likely to be referred to tertiary centers for work‐up and management.

Unlike similar studies with more homogenous patient populations, in this analysis we did not attempt to define the “optimal” treatment. In the absence of clinical context, such a determination is difficult to make. This is especially challenging for Tis/T1/Ta disease in the early stage group. The standard of care includes TURBT followed by either observation or adjuvant intravesicular therapy, then either observation or further surgery pending response to treatment.^18^ Since response to initial therapy is not captured by NCDB, and because several management options are recommended per NCCN, we did not identify “optimal” therapy. Furthermore, there may be several reasonable options in certain scenarios that cannot be ascertained using the NCDB, and “optimal” therapy may not be a consensus among clinicians even with complete clinical context. Nevertheless, in the aggregate, the NCCN‐sanctioned treatments were not executed as frequently with Black patients compared to non‐Black patients. It should be noted that the absolute difference was small: only 1.2% more white patients than Black patients were offered an acceptable treatment, per NCCN recommendations. However, once further investigated stage‐by‐stage in the propensity‐matched multivariable regression model, there is a glaring racial disparity, with Black patients being anywhere from 1.5–2 times less likely to receive an NCCN‐listed treatment in non‐metastatic disease. Black patients were also less likely to receive chemotherapy or radiation with metastatic disease; albeit this discrepancy was of more limited statistical significance.

The most comparable study to address this particular disparity is another NCDB analysis by Marinaro et al., which investigated solely muscle‐invasive disease and strictly defined the combination of neoadjuvant chemotherapy followed by cystectomy as “optimal treatment.” They reported an alarming odds ratio of 0.15 (95% CI 0.05–0.48) for Black patients receiving this particular treatment paradigm. While this is consistent with the most significant odds ratio in our analysis (OR = 0.51 in muscle invasive disease) for receipt of NCCN‐listed treatment by Black patients, our results are considerably different. There are several possible reasons for this, but namely, we included bladder preservation with chemoradiation (as suggested by the NCCN) as an acceptable treatment, whereas Marinaro and colleagues only identified neoadjuvant chemotherapy followed by cystectomy as appropriate management. The authors cite American Urology Association guidelines to justify this endpoint^27^; however, there are data to suggest that bladder preservation in select patients is more desirable with similar outcomes.^28^, ^29^ Nevertheless, when taken together, our studies consistently indicate that Black patients are disproportionately managed with bladder preservation for muscle‐invasive disease. Of note, unlike in this study, cases in the Marinaro study were propensity‐matched based on gender and not race, which may also further explain some of the discrepancies in our results.

With respect to gender, our study found similar but not identical results as it pertains to disparities in treatment selection compared to the Marinaro analysis. After propensity matching based on gender, the authors concluded that there was no difference in selection for optimal treatment (neoadjuvant chemotherapy followed by cystectomy) between men and women with muscle invasive bladder cancer. Conversely, we found that women were slightly less likely to receive an NCCN‐endorsed treatment compared to men at every stage of disease. However, the degree of disparity was not particularly strong, and certainly less strong compared to race. As demonstrated in Table 3, except for race and gender, no other demographic characteristics correlated with treatment disparities for each stage of disease. As one might expect, treatment at a community cancer center and uninsured/rural/lower income patients were less likely to receive a recommended treatment in some stages, but there was no consistent pattern. This further highlights the distinguished role race plays with respect to disparities in bladder cancer.

Another well described component of racial disparities in cancer management includes treatment delays, which have been reported for Black patients in breast, prostate, and lung cancer, among others.^16^, ^30^, ^31^ Interestingly and perhaps unexpectedly, no such racial inequities were noted in this analysis. In fact, the only demographic factors that independently correlated with a longer time from diagnosis to the start of treatment were treatment at community cancer centers and in rural areas. That is not to suggest that delays in overall medical care might not contribute to disparities in bladder cancer, as certainly the stark differences in stage at diagnosis may be partially due to delays in diagnosis. However, once patients were diagnosed, very few social inequities and none with respect to race were identified in this current analysis. This is in contrast to stage at diagnosis, where nearly every demographic characteristic independently correlated with a different stage at diagnosis, suggesting that social disparities in bladder cancer have more profound association with, if not impact on, bladder cancer detection rather than management.

Like all large retrospective studies, this analysis is subject to an inherent selection bias. It should be noted, however, that the data provided by this study can never manifest from a randomized study. Although several appropriate statistical measures were made to account for observable confounding variables such as multivariable regression analyses and propensity‐score matching, it is not possible to account for the unobservable confounding variables. For instance, smoking is a known risk factor of bladder cancer and is not captured by the database. The NCDB also does not capture the presentation of symptoms, so our assessment of stage at diagnosis does not necessarily represent when a patient presents with disease but rather when they have a histological confirmation of urothelial carcinoma. As mentioned in the methods, the “appropriate treatment” may be defined differently by different clinicians and, therefore, a similar analysis may produce somewhat incongruent results with variable definitions of treatment. We intentionally defined treatments broadly because they were mentioned as appropriate options by the NCCN, and to highlight the disparities revealed with respect to management even with widely inclusive definitions of treatment.

## 5 CONCLUSION

In the largest and most comprehensive study of its kind, we unveil the disappointing association between Black patients, late diagnosis, and non‐standard therapies for bladder cancer. While race was not the only demographic characteristic driving inequities in diagnosis and management, it appeared to be the most consistent and most significant. Female and uninsured/Medicaid patients also have later stage presentations and receive substandard treatment. Further investigation is warranted to reveal why such disparities exist and what can be done to mitigate them. We find it noteworthy that although disparities in bladder cancer treatments were evident, there was a stronger correlation between social factors and late diagnosis than with mismanagement or treatment delay. Therefore, addressing social inequities before bladder cancer diagnosis is made may have the greatest impact on alleviating the inequities in outcome.

## AUTHOR CONTRIBUTIONS

Concept and study analysis: Shaakir Hasan, Daniel Gorovets, Charles Simone. Writing manuscript: Shaakir Hasan, Stanislav Lazarev, Robert Press, Daniel Gorovets. Manuscript revision: Madhur Garg, Keyur Mehta, Arpit Chhabra, Isabelle Choi, Charles Simone.

## CONFLICT OF INTEREST

There was no funding involved in this research. None of the authors have any financial disclosures or conflicts of interest pertinent to this project. Given its retrospective nature and de‐identified dataset, this study was exempt from institutional review board approval. For the same reason, there were no direct human participants or patient material involved in this study requiring informed consent.

## Data Availability

Source data is produced from the National Cancer Database and can be obtained with permission from the American College of Surgeons.
